# Comparison of head tilt test between sagging eye syndrome and acquired unilateral trochlear nerve palsy

**DOI:** 10.1007/s00417-023-06347-z

**Published:** 2023-12-23

**Authors:** Katsuhide Yamadera, Akiko Kimura, Yoichi Okita, Yoshihito Mochizuki, Fumi Gomi

**Affiliations:** Department of Ophthalmology, Hyogo Medical University, 1-1, Mukogawa-Cho, Nishinomiya-City, Hyogo Japan

**Keywords:** Sagging eye syndrome, Trochlear nerve palsy, Differential point, Head tilt, Bielschowsky head tilt test

## Abstract

**Purpose:**

To investigate the distinction between sagging eye syndrome (SES group) and acquired unilateral trochlear nerve palsy (Trochlear group) in the Bielschowsky head tilt test (BHTT).

**Methods:**

Fifteen patients in the SES group (mean age 74.6 ± 5.2 years) and 14 patients in the Trochlear group (55.2 ± 15.9 years) visited the Department of Ophthalmology, Hyogo Medical University Hospital between November 2016 and October 2022 for treatment of their diplopia. Eye position was measured with the alternate prism cover test, and values for fixation of the dominant eye, or unaffected eye, were used. Cyclodeviation was measured with the synoptophore and the Glaucoma Module Premium Edition of the SPECTRALIS optical coherence tomography. In the BHTT, eye position was measured in three head postures: primary position (PP), head tilt to the side with hypertropia (Hyper), and head tilt to the side with hypotropia (Hypo). The differences in vertical deviation between PP and Hyper (Hyper − PP), PP and Hypo (PP − Hypo) and Hyper − Hypo were measured and compared.

**Results:**

Vertical deviation in primary position was 7.3 ± 4.5 PD in the SES group and significantly larger (17.1 ± 8.4 PD) in the Trochlear group (*p* = 0.002). The vertical deviation in Hyper was significantly larger in the Trochlear group with 7.7 ± 4.7 PD and 22.1 ± 9.4 PD, respectively (*p* < 0.001), whereas the that in Hypo was not significantly different between the two groups with 6.5 ± 3.4 PD and 8.4 ± 6.6 PD, respectively (*p* = 0.725). The SES group showed no significant difference according to the 3 head postures (*p* = 0.311), while the Trochlear group showed a significantly different with smaller mean values in vertical deviation in Hypo (*p* < 0.001). The difference in the vertical deviation for the 3 head postures was the largest in Hyper − Hypo (1.7 ± 2.1 PD and 13.6 ± 7.1 PD, respectively), and the accuracy of SES was at the cutoff value of 6 PD, and it was considered not to be SES if the value was 6PD or higher. The accuracy of SES determination was 100% sensitivity and 100% specificity, and the area under the curve was 1.0.

**Conclusion:**

The difference in Hyper − Hypo in the BHTT may be the most useful index in differentiating SES from acquired unilateral trochlear nerve palsy; if the difference was more than 6 PD, the probability of SES was very low.


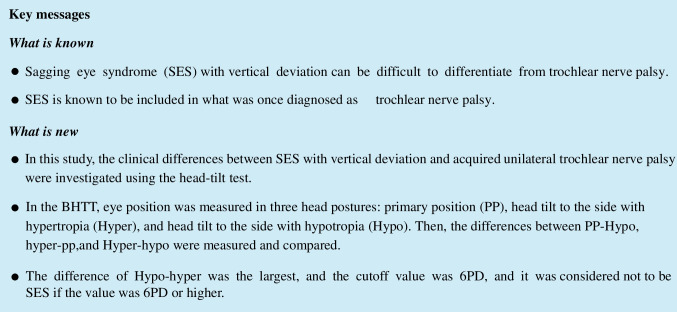


## Introduction

Acquired trochlear nerve palsy, which is often observed after microcirculatory disturbance or trauma, has been considered to account for most cases of vertical strabismus and cyclotorsion [1]. However, in 2013, Chaudhuri and Demer reported “sagging eye syndrome” (SES), caused by age-related changes in the orbital pulley [2], and it was pointed out that SES is often the cause of vertical strabismus and cyclotorsion that had been considered to be caused by trochlear nerve palsy [2, 3]. SES is caused by rupture of the lateral rectus (LR) – superior rectus (SR) band or bowed LR with inferior displacement of the LR due to aging and is characterized by age-related distance esotropia (ARDE) in bilateral cases and small angle cyclo-vertical strabismus (CVS) in unilateral cases. The latter presents clinical findings similar to those of acquired unilateral trochlear nerve palsy and is often difficult to distinguish between CVS-type SES (SES group) and acquired unilateral trochlear nerve palsy (Trochlear group).

Wei et al. reported the three-step test is often positive in SES clinical alignment patterns may confound SES with acquired superior oblique palsy [4].

In this study, we investigated the clinical differences between SES group and Trochlear group using a head-tilt test, in addition to evaluation of horizontal deviations, vertical deviations and cyclotorsion, to determine the relevant clinical differences.

## Methods

Of the patients with vertical strabismus and/or cyclotorsion who visited our department between November 2016 and October 2022 for the elimination of diplopia, 15 patients in the SES group (mean age 74.6 ± 5.2 years, 7 males and 8 females) and 14 patients in the Trochlear group (mean age 55.2 ± 15.9 years, 8 males and 6 females) met the following diagnostic criteria.

**Fig. 1:**
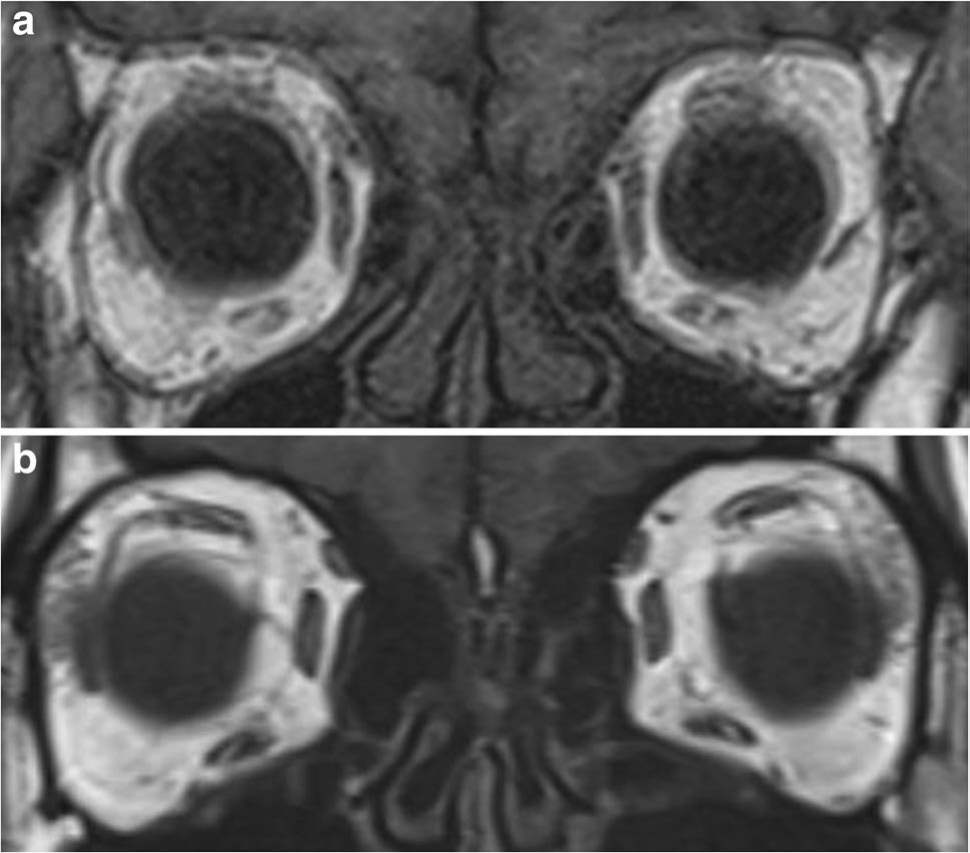
Orbital MRI. **a** A typical SES; MRI coronal section showed rupture of the LR-SR band and the temporally tilting of superior part of the LR only on the left eye. **b** A typical Trochlear nerve palsy; MRI coronal section did not show SO atrophy, LR-SR band rupture and LR inferior displacement

The diagnosis of SES was made by magnetic resonance imaging (MRI) in all patients (Fig. 1a), which showed inferior displacement of the LR, rupture of the LR-SR band or bowed LR. In addition, axial MRI demonstrated that the LR was markedly elongated in SES, and SO on cross section of MRI were normal in CVS [5]. ARED is present when the inferior displacement of LR pulley was bilaterally symmetrical. The characteristic facial features of SES were defined as a superior sulcus deformity, aponeurotic blepharoptosis, blepharoptosis, and baggy lower eyelid [6].

The diagnosis of unilateral trochlear nerve palsy was made after trauma or acute onset, with subjective symptoms of vertical or cyclo-diplopia in primary position (PP), which were aggravated by downward, unilateral inferior oblique muscle overaction and superior oblique muscle under-action, and no abnormal findings in extraocular muscles on MRI (Fig. 1b). To exclude thyroid ophthalmopathy and myasthenia gravis, thyroid-related autoantibodies and anti-acetylcholine receptor antibodies were measured, and those positive for either were excluded. Patients with diurnal variation were also excluded.

The angle of deviation was measured with the alternate prism cover test (APCT) in the PP, and the data of APCT for distance was used. The data of strabismus angle were those of dominant eye fixation in the SES group and those of the unaffected eye fixation in the Trochlear group. The vertical deviations were measured by head tilt to the hypertropia side (Hyper) and to the hypotropia side (Hypo), according to Bielschowsky head tilt test (BHTT), and the differences in vertical deviation between PP and Hyper, PP and Hypo, and Hyper and Hypo were calculated and compared between the groups. Subjective cyclotorsion was measured using the cross slide of a synoptophore, and the sum of the two eyes was used for the objective cyclotorsion which was measured, one eye at a time, with the Glaucoma Module Premium Edition (GMPE) of the SPECTRALIS OCT (Heidelberg) [4]. We used the Mann–Whitney test to test between the two groups, and The Friedman test between the three groups, and *p* < 0.05 was considered significant. If the Friedman test showed no significant difference in the SES group, but only in the Trochlear group, the Bonferroni method was used to examine significant differences between the groups for the Trochlear group only. And receiver operating characteristic (ROC) curves were drawn, and the sensitivity and specificity of diagnostic indicators were calculated and added to the method.

## Results

**Fig. 2:**
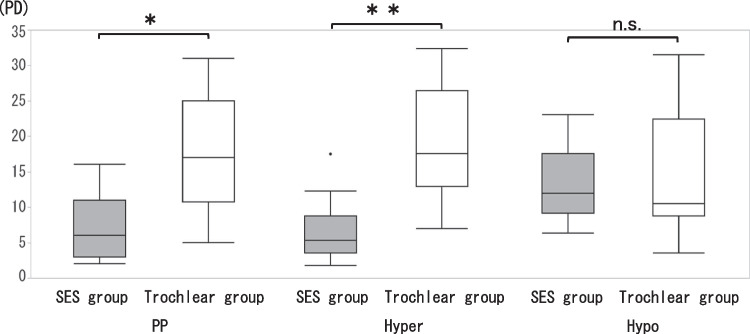
Vertical deviation for three head positions. In PP and Hyper, there was a significant difference between the SES group and the Trochlear group (*: *p* = 0.002, **: *p* < 0.001), but no significant difference in Hypo (*p* = 0.725)

**Fig. 3:**
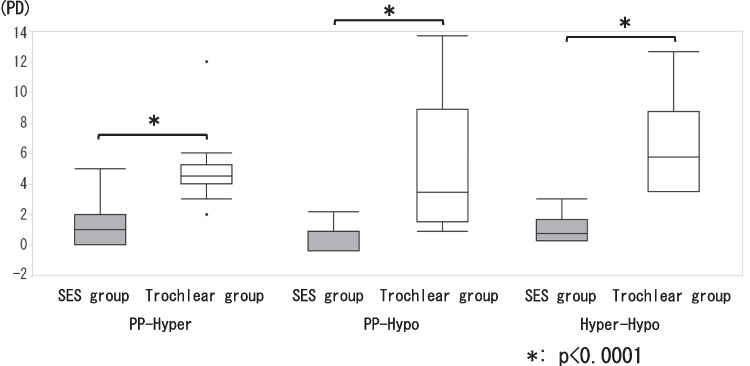
Vertical deviation between PP − Hyper, PP − Hypo, and Hyper − Hypo. The difference in vertical deviation between the SES and Trochlear groups in all three head positions was significant(*p* < 0.001 for each direction), but the largest difference was Hyper − Hypo (SES group: 1.7 ± 2.1 PD, Trochlear group: 13.6 ± 7.1 PD)

**Fig. 4:**
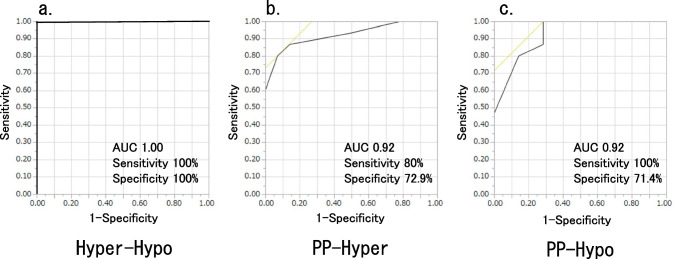
ROC analysis

**Fig. 5:**
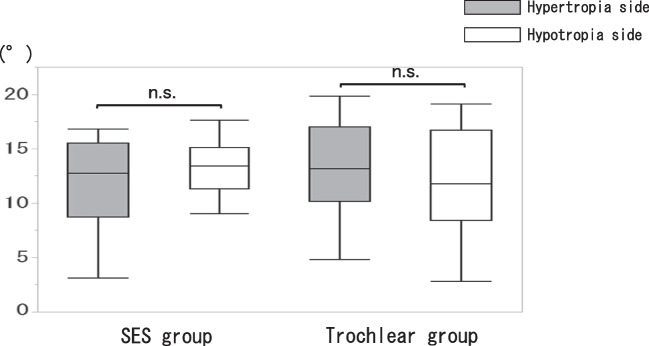
Cyclotorsion of the eye with hypertropia and hypotropia by GMPE. Neither the SES group nor the Trochlear group showed significant differences in the cyclotorsion of the eye with hypertropia and hypotropia (*p* = 0.443, *p* = 0.491)

I. Horizontal deviation
  In the SES group, 4 (26.7%) patients had orthophoria, 9 (60.0%) had esotropia (mean angle: 7.0 ± 5.1 PD) and 2 (13.3%) had exotropia (7.0 ± 1.4 PD). In contrast, one (7.1%) patient in the Trochlear group had orthophoria, 3 (21.4%) had esotropia (3.0 ± 2.6 PD) and 10 (71.4%) had exotropia (6.8 ± 5.2 PD). Horizontal deviation showed a significant tendency of esotropia in the SES group (*p* = 0.005).
II. Vertical deviation
  ➀ The vertical deviations of the SES and Trochlear groups in the PP, the SES group showed 7.3 ± 4.5 PD and the Trochlear group 17.1 ± 8.4 PD, which was significantly larger in the Trochlear group (*p* = 0.002).
  ➁ The vertical deviations of the SES and Trochlear groups in the 3 head postures were shown in Fig. 2. For the Hyper, the Trochlear group was significantly larger with 7.7 ± 4.7 PD and 22.1 ± 9.4 PD, respectively (*p* < 0.001) and 6.5 ± 3.4 PD and 8.4 ± 6.6 PD for the Hypo respectively, showing no significant difference between the two groups (*p* = 0.725). In the SES group, no significant difference was found for 3 head postures (*p* = 0.311), and in the Trochlear group, Hypo was significantly smaller (*p* < 0.001, PP vs Hyper: *p* = 0.589, PP vs Hypo: *p* = 0.018, Hyper vs Hypo: *p* = 0.002).
    The differences in vertical deviation in the 3 head postures by BHTT was 1.5±1.6 PD in the SES group and 4.9±2.3 PD in the Trochlear group for PP−Hyper, 1.6±1.4 PD and 8.7±7.0 PD for PP−Hypo, and 1.7±2.1 PD and 13.6±7.1 PD for Hyper−Hypo, respectively (Fig. 3). Vertical deviation was significantly larger in the Trochlear group in all head postures (p<0.001 for each head positions), with the largest difference in Hyper−Hypo.
  ➂ The cutoff values for the Trochlear and SES groups were 11 PD of vertical deviation for PP and 6 PD for Hyper-Hypo. With the cutoff value of 11 PD, the values of sensitivity, specificity and area under the curve (AUC) were 80%, 58.6% and 0.84, respectively, for the accuracy of determining that the vertical deviation in the PP was due to SES. With a cutoff of 2 PD, the sensitivity, specificity and AUC of PP − Hyper were 80%, 72.9% and 0.92, respectively. The corresponding values for PP-Hypo were 100%, 71.4%, and 0.92, respectively, with the cutoff value of 4 PD. Hyper − Hypo had a sensitivity of 100%, specificity of 100%, and AUC of 1.0 with a cutoff value of 6 PD (Fig. 4).
III. Cyclotorsion
  ➀ For the synoptophore, there was no significant difference between the SES group (7.5 ± 2.5°) and the Trochlear group (8.3 ± 3.2°) (*p* = 0.568). Likewise, there was no significant difference between the SES group (22.9 ± 5.2°) and the Trochlear group (24.5 ± 6.4°) for the GMPE (*p* = 0.266).
  ➁ The cyclotorsion of the hypertropia and hypotropia side on GMPE were 11.7 ± 4.5° and 13.4 ± 2.5°in the SES group (*p* = 0.443) and 12.7 ± 4.4° and 11.8 ± 5.0°in the Trochlear group (*p* = 0.491), respectively, showing no significant difference between the two side (Fig. 5).

## Discussion

In 1995, Demer et al. reported that connective tissue pulleys histologically and functionally control the movement of the rectus muscles [7, 8]. Furthermore, they reported that there is a correlation between the restriction of vertical eye movement and the sagging of the horizontal rectus muscle pulley, that the pulley affects the horizontal, vertical and cyclo-movements by all extraocular muscles, and that connective tissue degeneration occurs with aging [9–11]. In 2013, this strabismus caused by age-related connective tissue degeneration was named “sagging eye syndrome” [2]. SES has two types (ARDE and CVS), with the identification of SES, many cases of vertical strabismus and/or cyclotorsion of unknown etiology, were found to be caused by SES [3]. On the other hand, acquired unilateral trochlear nerve palsy is often difficult to differentiate from SES when it is not caused by trauma or intracranial lesions. In acquired trochlear nerve palsy caused by microcirculatory disturbance, MRI does not show atrophy of the superior oblique muscle, but only almost age-appropriate changes. Furthermore, since it has been reported that the LR-SR band is elongation in approximately 50% of elderly patients without strabismus [2], it is possible that patients with acquired trochlear nerve palsy also show rupture or elongation of LR-SR band. Regarding differential point, it has been reported that SES is characterized by exocyclotorsion in the eye with hypotropia because the eye with greater sagging of LR becomes hypotropia when the configuration of LR-SR pulley is asymmetric between the two eyes [2, 3]. However, in this study, there was no significant difference in exocyclotorsion in either the SES or Trochlear group.

As in the previously reported [12–14], the horizontal deviation showed a tendency to be esodeviation in SES, while the vertical deviation showed a tendency to be small in SES. In primary position, the vertical deviation in SES seems to be smaller than that in the acquired trochlear nerve palsy, but since no cutoff value is given, it is difficult to distinguish between SES from acquired trochlear nerve palsy with small vertical deviation.

For a long time, Parks' 3-step method, which includes a head-tilt test, has been used to diagnose trochlear nerve palsy (superior oblique palsy: SOP) [1, 15]. This test is known to have a low positivity rate, especially in patients with atrophy of the SO muscle, and Demer et al. reported a positivity rate of 50% [16]. The 14 cases of acquired trochlear nerve palsy in this study did not show atrophy of the SO muscle on MRI, but Uchiyama, et al. [17] and Lee, et al. [18] reported the positive rates in cases without atrophy of the SO muscle to be 75% and 78%, respectively. Furthermore, it has been reported that about half of the SES cases fulfilled Parks' 3-step method [19]. Some reports define BHTT positively as a change of 1 PD or more [18, 20], while others define BHTT positively as a difference of 5 PD or more in vertical deviation by head tilt [21]. As previously reported, 6 of 15 patients (40%) in the SES group were positive when BHTT was defined as 1 PD or more in this study. Therefore, it is difficult to differentiate SES from acquired trochlear nerve palsy by BHTT alone.

Hence, we decided to compare the differences in vertical deviation of BHTT, between primary position (PP) and hypertropia side (Hyper), between PP and hypotropia side (hypo), and between Hyper and Hypo, thinking that these differences might be useful for differentiating between SES and acquired unilateral trochlear nerve palsy. In the SES group, head tilt did not significantly change the vertical deviation, but in the Trochlear group, head tilt to the Hypo resulted in a significantly smaller vertical deviation. The deviation of PP − Hypo, PP − Hyper, and Hyper − Hypo were all significantly smaller in the SES group than in the Trochlear group, but the most obvious difference between the two groups was found in Hyper − Hypo.

In the ROC analysis logistic regression of Hyper − Hypo, which showed the largest difference between the SES and Trochlear groups in this study, the sensitivity was 100%, specificity was 100%, and AUC was 1.0 at the cutoff value of 6 PD, indicating that SES with a Hyper − Hypo value of 6 PD or more is extremely rare.

The limitation of this study was the small number of samples. The sensitivity and specificity of 100% were probably due to the small number of samples (Fig. 4).

In this study, even without MRI, the difference between Hyper and Hypo may be the most useful index for differentiating SES and acquired unilateral trochlear nerve palsy.
